# The Potential Regulatory Roles of lncRNAs in DNA Damage Response in Human Lymphocytes Exposed to UVC Irradiation

**DOI:** 10.1155/2020/8962635

**Published:** 2020-03-13

**Authors:** Dan Xu, Yue Wang, Jia Wang, Fei Qi, Yeqing Sun

**Affiliations:** Institute of Environmental Systems Biology, College of Environmental Science and Engineering, Dalian Maritime University, Dalian, Liaoning, Linghai Road 1, 116026, China

## Abstract

Long noncoding RNAs (lncRNAs) are a class of noncoding RNAs that modulate gene expression, thereby participating in the regulation of various cellular processes. However, it is not clear about the expression and underlying mechanism of lncRNAs in irradiation-induced DNA damage response. In the present study, we performed integrative analysis of lncRNA-mRNA expression profile in human lymphocytes irradiated with ultraviolet-C (UVC). The results showed that exposure to UVC irradiation dose-dependently increased the fluorescence intensity of *γ*-H_2_AX and induced cell death. Microarray analysis revealed that up-regulated lncRNAs were more common than down-regulated lncRNAs with the increase of radiation dose in UVC-radiated cells. Stem analysis demonstrated the relationship between lncRNA expression level and radiation dose. qPCR results confirmed that LOC338799 and its coexpressed genes such as LCE1F and ISCU showed the increase in expression levels with the increase of UVC radiation dose. We utilized Cytoscape to screen out 5 lncRNAs and 13 coexpressed genes linking to p53, which might participate in the regulation of DNA damage, cell cycle arrest, apoptosis, and cell death. These findings suggest that lncRNAs might play a role in UVC-induced DNA damage response through regulating expression of genes in p53 signaling pathway.

## 1. Introduction

Ultraviolet (UV) radiation belongs to the nonionizing part of the electromagnetic spectrum, which is subdivided into UVA (wavelengths 315-400 nm), UVB (280-315 nm), and UVC (200-280 nm). Recently, UVC radiation gained more attention because UVC has direct damaging effects to cellular DNA and results in DNA single- and double-strand breaks [1, 2]. Cellular DNA has a higher absorption peak at 260 nm within the UVC band, so UVC is often used as the easiest and fastest way to produce DNA damage. UVC can induce immediate DNA damage and energy-dependent biological effects in a variety of cells. It is reported that UVC radiation-induced DNA damage provoked highly divergent responses in human skin fibroblasts exposed to low (10 J/m^2^) and high doses (50 J/m^2^) of UVC radiation [3]. Another report showed that UVC could affect the transcriptional profile in human primary cultured fibroblasts irradiated with even a low dose (0.5 or 5 J/m^2^) of UVC [4]. Cell viability and apoptosis of mouse embryonic fibroblasts were investigated after exposure to three doses (50, 75, and 300 J/m^2^) of UVC radiation [5].

When cells are exposed to environmental stress, DNA damage often occurs and subsequently causes DNA-damage response (DDR) including cell cycle arrest, apoptosis, and DNA repair [6]. DNA damage can also result in cell death unless it is repaired or tolerated [7]. TP53 is one of the major regulators in DDR after irradiation, which can activate many downstream genes including p21WAF1/CIP1 (CDKN1A), Bcl-2-Associated X Protein (BAX), Bcl-2-Binding Component 3 (BBC3), Cyclin Dependent Kinase 6 (CDK6), DNA Damage 45 alpha (GADD45A), and Late Cornified Envelope Group I (LCE1) family [8–12]. It is reported that most of p53 target genes together construct the p53 network in response to irradiation [13, 14]. Therefore, p53 signaling pathway is considered to be the most important pathway and a number of genes have been suggested to become potential radiation dosimeters [15].

Long noncoding RNAs (lncRNAs) are defined as RNA molecules longer than 200 nucleotides in length, which can modulate gene expression through a variety of mechanism [16]. A number of reports revealed that lncRNAs could affect not only gene expression but also protein translation and stability [17]. LncRNAs are less conserved in sequence and only about 12% of lncRNAs can be found other than humans. Deep sequencing recently has been utilized to discover novel lncRNAs. LincRNA-p21 is first identified as a direct transcriptional target of p53. The induction of lincRNA-21 by UVB irradiation was primarily through a p53-dependent pathway and had a proapoptotic function in keratinocytes [18]. It is reported that HULC promoted UVB-induced cell injury via the activation of JAK/STAT (1/3) signaling pathway in HaCaT cells [19]. HOTAIR resulted in apoptosis and inflammation in UVB-exposed keratinocytes [20]. However, it is not clear about the expression and underlying mechanism of lncRNAs in DDR induced by UVC irradiation.

Human lymphocytes have been widely used in the field of radiation research about DNA damage. We select human CD4^+^ T lymphocytes (CD4) because they are nucleated cells and easily separated from human blood. Here, we aimed to study the dose-dependent expression changes of lncRNAs in CD4 cells exposed to UVC irradiation. We performed coexpression network analysis of lncRNA-mRNA and revealed that novel lncRNAs might play a crucial role in DDR induced by UVC through p53 signaling pathway. Our study will provide important experimental guide for screening lncRNAs as new radiation dosimeters in the future.

## 2. Materials and Methods

### 2.1. Cell Culture and UVC Radiation

The human CD4^+^ T lymphocytes (CD4) (ATCC, Manassas, VA) were cultured in RPMI 1640 Medium (GIBCO, Carlsbad, CA) containing 10% fetal bovine serum (GIBCO) and antibiotic in an incubator. CD4 cells were cultured in T25 flasks and passaged every 4 days.

CD4 cells were suspended in 1 ml culture medium, evenly covering with the bottom of 60 mm petri dish. The lid was open when cells were irradiated with 4-64 J/m^2^ UVC light (0.11 J/m^2^/s at 254 nm) in a dark box. Control cells were treated similarly with the exception that they did not undergo UVC irradiation. Subsequent to UVC exposure, the cells were placed in an incubator for indicated time until their use.

### 2.2. *γ*-H_2_AX Fluorescence Assay

CD4 cells were untreated or radiated with UVC at different doses, and then cultured in an incubator for 30 min. Phosphorylated histone H_2_AX (*γ*-H_2_AX) was detected by *γ*-H_2_AX Fluorescence Assay Kit according to the manufacturer's instructions (Upstate Biotechnology Inc., NY, USA). In brief, the cells were collected, fixed, permeabilized, and stained with FITC-conjugated antiphospho-histone H_2_AX (Ser139). Cells are then scanned in a flow cytometer (BD Biosciences, San Jose, CA) to quantitate the number of cells staining positive for *γ*-H_2_AX. The relative fluorescence intensity was used to reflect the appearance of *γ*-H_2_AX in comparison to nonradiated samples.

### 2.3. Quantitative PCR (qPCR)

Total RNA was extracted using the TRIzol reagent. The expression levels of mRNAs and lncRNAs were quantified by qPCR. Real-time PCR reactions were performed with SYBR Green Master Mix using a Light Cycler®48 II real-time PCR system (Applied Biosystems, CA). The primer sequences in qPCR were listed in Table. [Supplementary-material supplementary-material-1]. The relative expression level was calculated using the comparative delta CT method (2^-△△Ct^) after normalization with reference to the expression of GAPDH.

### 2.4. LncRNA-mRNA Microarray Analysis

After UVC irradiation, CD4 cells were placed in an incubator for 24 hours and then used for microarray analysis using Affymetrix Human HTA2.0. LncRNA-mRNA microarray analysis was supplied by Shanghai OE Biotechnology Company. The sample treatments were based on the manufacturer's standard protocols. The expression level of each lncRNA or mRNA was presented as fold change. Differentially expressed lncRNAs or mRNAs (≥2-fold) were identified to be significant. A filtering step was applied to reduce the number of multiple hypotheses. Only those genes annotated with NM_numbers or ENST-numbers, and those lncRNAs annotated with NR_numbers or ENST-numbers, were included in the final analysis.

### 2.5. Stem Analysis and Coexpression Analysis of lncRNA-mRNA

Stem analysis and coexpression analysis for lncRNA-mRNA were supplied by Shanghai OE Biotechnology Company. In stem analysis, differentially expressed genes or lncRNAs were divided into 30 categories and the upper left-hand digit of each small graph was the category number. No. 21 and No. 4 had significant differences (fold changes ≥2 and *P* ≤ 0.05), whereas the others had no significant differences.

The Pearson correlation between expression value of each lncRNA and expression value of its coexpressed mRNA was calculated. When *P* value of the coefficient correlation was not higher than 0.05 and the absolute value of correlation was not less than 0.7, they were considered to be relevant. The top 30 coexpressed mRNAs of each lncRNA were selected to discuss the regulatory relationship between lncRNA and coexpressed mRNA using Cytoscape 3.6.1 (https://cytoscape.org/) [21].

### 2.6. Statistical Analysis

All data are presented as means ± standard deviations (SD). Regression analysis and Student's *T*-test analysis were performed using SPSS version 17.0. *P* < 0.05 were considered statistically significant.

## 3. Results

### 3.1. Effect of UVC Irradiation on DNA Damage and Cell Death in CD4 Cells

**Figure 1:**
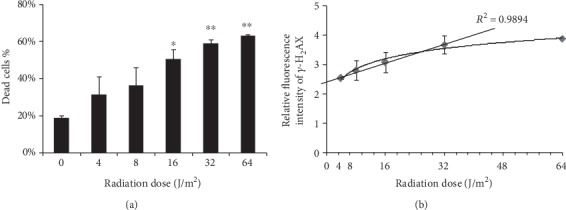
The effects of UVC on DNA damage and cell death in CD4 cells. (a) The percentage of dead cells was calculated in CD4 cells at 24 h after UVC irradiation. (b) The relative fluorescence intensity of *γ*-H_2_AX was examined in CD4 cells at 30 min after UVC irradiation. The straight line indicates a linear relationship between the fluorescence intensity of *γ*-H_2_AX and the radiation dose within the dose range of 4-32 J/m^2^. The results are displayed as the mean ± SD (*n* = 3). ^∗^*P* < 0.05, ^∗∗^*P* < 0.01 compared with the control group.

We firstly identified that the optimal radiation dose range of UVC was 4-64 J/m^2^, within which the percentage of dead cells was 30-70% in CD4 cells at 24 h after UVC irradiation. UVC irradiation caused cell death in a dose-dependent manner, showing the significant increase in the percentage of dead cells in the 16, 32, and 64 J/m^2^ groups compared with the control group (Figure 1(a)). We then examined relative fluorescent intensity of *γ*-H_2_AX in CD4 cells at 30 min after UVC radiation. The results showed that UVC radiation increased relative fluorescent intensity of *γ*-H_2_AX in a dose-dependent manner. There was a linear relationship (*R*^2^ = 0.9894) between the fluorescence intensity of *γ*-H_2_AX and the radiation dose within the dose range of 4-32 J/m^2^ (Figure 1(b)).

### 3.2. Effect of UVC Radiation on Differentially Expressed mRNA and lncRNAs

**Figure 2:**
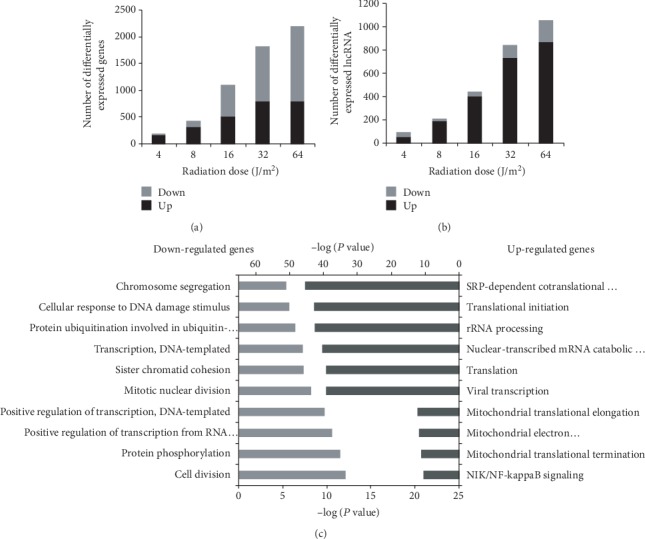
Microarray analysis of gene and lncRNA expression profiles under UVC irradiation. (a, b) The number of differentially expressed genes (a) and lncRNAs (b) was shown in the five radiation dose groups. (c) Function analysis of down-regulated genes and up-regulated genes.

We performed microarray analysis of gene and lncRNA expression profiles. The results showed that UVC radiation induced the increase in the number of differentially expressed genes and lncRNAs (≥2-fold) in a dose-dependent manner (Figures 2(a) and 2(b)). The number of 2-5-fold up- or down-regulated genes (Table. [Supplementary-material supplementary-material-1]) and lncRNAs (Table. [Supplementary-material supplementary-material-1]) was listed in detail. We observed that there were much more up-regulated genes in lower dose groups and much more down-regulated genes in higher dose groups (Figure 2(a)). In contrast, most of lncRNAs were up-regulated in all UVC-radiated groups (Figure 2(b)). GO analysis showed that most of down-regulated genes function on cell division, protein phosphorylation, transcription, and cellular response to DNA damage stimulus. Those up-regulated genes may be involved in various translation processes (Figure 2(c)).

### 3.3. Relationship Analysis between Expression Alteration and Radiation Dose

**Figure 3:**
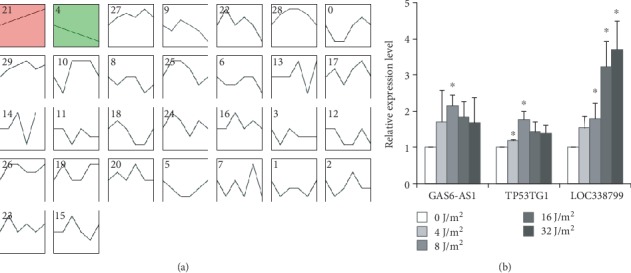
Stem analysis and validation of lncRNA expression alterations. (a) Differentially expressed lncRNAs are divided into 30 categories, and the category number No. 21 represents the expression of lncRNAs increased significantly with the increase of UVC radiation dose, No.4 represents the expression of lncRNAs decreased significantly with the increase of UVC radiation dose. (b) qPCR results confirmed the expression alterations of three lncRNAs in CD4 cells at 24 h after UVC irradiation. ^∗^*P* < 0.05 compared with control group.

To observe the relationship between expression alteration of gene or lncRNA and radiation dose, we performed stem analysis. The results showed that the expression of 729 genes and 797 lncRNAs increased significantly with the increase of UVC radiation dose whereas 1372 genes and 133 lncRNAs showed the significant decrease in expression levels with the increase of UVC radiation dose (Fig. [Supplementary-material supplementary-material-1] and Figure 3(a)).

UV radiation is an environmental hazard and mutagen, leading to an increased risk of human cancers. We utilized lncRNA disease database, and found that three lncRNAs including GAS6 antisense RNA 1 (GAS6-AS1), TP53 target 1 (TP53TG1), and Telomerase RNA component (TERC) were known to be associated with human cancers [22–24]. qPCR results confirmed the up-regulation of GAS6-AS1 and TP53TG1 in 4-32 J/m^2^ dose groups (Figure 3(b)), although the expression of TERC showed an increased trend with the increase of radiation dose without statistical significance (data not shown). Notably, the expression of LOC338799 increased significantly with the increase of UVC radiation dose whereas TP53TG1 expression showed the significant increase in 4 and 8 J/m^2^ dose groups.

### 3.4. Coexpression Network Analysis of lncRNA-mRNA

**Figure 4:**
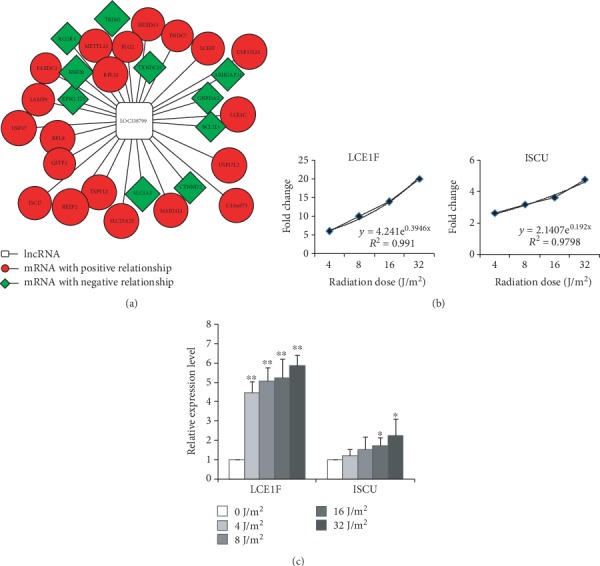
Regulation network of LOC338799 and coexpressed mRNAs. (a) LOC338799 has 30 coexpressed genes, including 20 positively regulated genes (red) and 10 negatively regulated genes (green). (b) Regression analyses of coexpressed genes LCE1F and ISCU show dose-dependent relationships between gene expression alteration and the radiation dose within the dose range of 4-32 J/m^2^. (c) qRT-PCR results confirmed the expression alterations of LCE1F and ISCU in CD4 cells at 24 h after UVC irradiation. ^∗^*P* < 0.05, ^∗∗^*P* < 0.01 compared with the control group.

Each lncRNA had a positive or negative regulation relationship with coexpressed gene. We searched for some lncRNAs such as LOC338799, TERC, and USP17L6P from significantly up-regulated lncRNAs with the increase of UVC radiation dose, based on the results from stem analysis. We focused on LOC338799, which had positive regulation relationship with 20 genes while it had negative regulation relationship with 10 genes (Figure 4(a)). The up-regulation of LCE1F and ISCU showed an exponential trend with the increase of radiation dose (Figure 4(b)). qPCR results confirmed the up-regulation of LCE1F and ISCU in a dose-dependent manner within the dose range of 4-32 J/m^2^ (Figure 4(c)). Similarly, we found that TERC had a positive regulation relationship with 15 genes such as apoptotic regulator 1 (MOAP1) and eukaryotic translation initiation factor 3 subunit D (EIF3D) that were reported to regulate cell cycle, apoptosis, and cell death [25, 26]. USP17L6P had a positive regulation relationship with TP53-regulated inhibitor of apoptosis (TRIAP1) that was associated with apoptosis and cell death [27].

**Figure 5:**
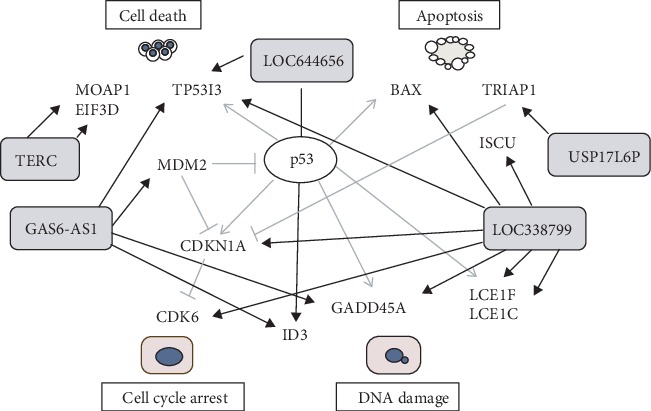
Regulatory network diagram of lncRNAs and coexpressed genes linking to p53. Regulatory network diagram of 5 lncRNAs and 13 coexpressed genes linking to p53 were constructed. They together play important roles in UVC-induced DDR including DNA damage, cell cycle arrest, cell death, and apoptosis.

Further, Cytoscape were utilized for coexpression network analysis of lncRNA-mRNA. We wonder how many coexpressed genes of lncRNAs may be regulated by p53. We screened out 13 coexpressed genes of 5 lncRNAs, possibly involved in DDR via p53 signaling pathway (Figure 5). All the lncRNAs were up-regulated and most of the genes except CDK6 showed the increase in expression levels in UVC-radiated groups although the induction levels varied (Table 1). GAS6-AS1 had 4 coexpressed genes including GADD45A, MDM2, Tumor Protein P53 Inducible Protein 3 (TP53I3), and inhibitor of DNA binding 3 (ID3). LOC338799 had 8 coexpressed genes including GADD45A, TP53I3, CDK6, BAX, CDKN1A, LCE1C, LCE1F, and ISCU. TRIAP1 is coexpressed gene of USP17L6P. TP53I3 and ID3 are coexpressed genes of LOC644656. qPCR results confirmed the up-regulation of CDKN1A, GADD45A, and TRIAP1 within the dose range of 4-32 J/m^2^ (Fig. S2). Taken together, these genes may participate in the regulation of DDR including cell cycle arrest, DNA damage, cell death, and apoptosis.

## 4. Discussion

In the present study, we investigated the expression alterations of lncRNAs in human lymphocytes exposed to five doses of UVC radiation. We found that most of lncRNAs were up-regulated at 24 h after UVC irradiation. We performed lncRNA-mRNA coexpression network analysis, demonstrating the potential regulatory role of lncRNAs in UVC-induced DDR via p53 signaling pathway. Especially, LOC338799 and coexpressed genes such as LCE1F and ISCU showed the increase in expression levels with the increase of UVC radiation dose. We suppose that five candidate lncRNAs including LOC338799, LOC644656, GAS6-AS1, TERC, and USP17L6P could be involved in DNA damage, cell cycle, apoptosis, and cell death through the regulation of coexpressed genes.

We selected the optimal dose range of UVC radiation, based on determining the status of live and dead cells after UVC radiation. We identified the percentage of dead cells was 30-70% in CD4 cells at 24 h after 4-64 J/m^2^ of UVC irradiation. Therefore, the optimal dose range of UVC radiation was 4-64 J/m^2^ in our experimental settings. It is known that *γ*-H_2_AX is a highly specific and sensitive molecular marker for the detection of DNA damage. The detection of *γ*-H_2_AX foci in DNA strand break (DSB) sites or the increase in *γ*-H_2_AX fluorescent intensity indicates the occurrence of DNA damage [28]. We found that UVC increased the relative fluorescence intensity of *γ*-H_2_AX in a dose-dependent manner within the dose range of 4-32 J/m^2^ except 64 J/m^2^, suggesting that 4-32 J/m^2^ may be the optimal dose range to study the dose-dependent expression alterations and regulation mechanisms of lncRNAs in the further experiments.

PLK2 plays a key role in cell cycle progression, which is activated at the G1-S transition of cell cycle [29]. TRIAP1 overexpression inhibited cell death and apoptosis by inhibiting p21, which contributed to mitochondrial-dependent apoptosis resistance [27]. In this study, we found that PLK2 and TRIAP1 were up-regulated in UVC-irradiated CD4 cells and qPCR results confirmed the increase in their expression levels with the increase of UVC radiation dose (Fig. [Supplementary-material supplementary-material-1]). ISCU is the main scaffold protein for Fe-S cluster assembly, involved in the regulation of cell metabolism [30]. LCE1F belongs to LCE1 family, which are target genes of p53. LCE1F protein interacts with protein arginine methyltransferase 5 (PRMT5) in response to DNA damage [10]. MOAP1 participates in the internal and external pathways of cell death and activates the apoptotic protein BAX of Bcl-2 family [25]. Studies have shown that knockout of EIF3D significantly induces G2/M arrest and apoptosis by down-regulating cyclin B1 and up-regulating p21 [26].

Among the top 30 coexpressed genes of GAS6-AS1, GADD45A, MDM2, TP53I3, and ID3 have been reported to maintain genomic stability. GADD45A is known downstream gene of p53 and regulate many cellular processes such as DNA damage, cell cycle, and apoptosis [31, 32]. MDM2 is a key regulator of the expression and function of p53, which inhibits p53-mediated cell cycle arrest and apoptosis [33]. TP53I3 is induced by p53 and involved in p53-mediated cell death [34]. It is reported that knockout of ID3 resulted in a significant increase in DNA damage accumulation and chromosomal aberration. After ionizing radiation, ID3 is phosphorylated by ATM and MDC1, resulting in the recruitment of additional DDR factor at DSB sites [35].

In this study, we found that LOC338799 showed the increase in expression levels with the increase of UVC radiation dose. LOC338799 had 8 coexpressed genes, among which most of the genes showed the increased expression levels (≥2-fold) except LCE1C whereas CDK6 was down-regulated in a dose-dependent manner. qPCR results also confirmed positive relationship between LOC338799 and coexpressed genes such as ISCU and LCE1F. CDK6 promotes cell cycle progression from G1 to S, inhibited by CDKN1A [36]. BAX induces apoptosis as a direct transcriptional target of p53 [37]. LCE1C and LCE1F belong to LCE1 family as p53 downstream targets. It is reported that induction of LCE1 expressions was caused by UV irradiation in a p53-dependent manner and might have functions on DNA damage through modulation of the PRMT5 activity [10]. Taken together, LOC338799 might play a critical role in p53-mediated DDR through regulating the expression of its coexpressed genes.

It is known that some lncRNAs have been associated with human diseases, implicated in the progression of human cancer. GAS6-AS1 was down-regulated in 50 cases of non-small-cell lung cancer, negatively correlated with lymph node metastasis and advanced lymph node metastasis [22]. The expression of TP53TG1 was significantly increased in human glioma tissues or cell lines. In the case of sugar deficiency, TP53TG1 knockout decreased cell proliferation and migration [23].

Although the number of novel lncRNAs is increasing, cellular functions of many lncRNAs remain unknown. Our results suggest that specific lncRNAs might be involved in DDR induced by UVC irradiation via p53 signaling pathway. We also performed sequence analysis of lncRNA and coexpressed genes, and confirmed that there were the same short sequences (>10 nt) between some lncRNAs and their coexpressed genes (data not shown), indicating that they might work together to participate in DDR. Further, it is necessary to do knockout or overexpression experiments to understand their contributions to DDR in the near future.

## 5. Conclusions

In this study, we at the first time investigated the expression alterations of mRNAs and lncRNAs in CD4 cells after UVC radiation. We propose an integrated molecular mechanism of lncRNAs in UVC-induced DNA damage in human lymphocytes. LOC338799 showed the increase in expression levels with the increase of UVC radiation dose. LOC338799 and 8 coexpressed genes were most likely involved in the regulation of DNA damage, cell cycle, apoptosis, and cell death via p53 signaling pathway.

## Figures and Tables

**Table 1:** Coexpressed genes of lncRNAs function in DDR via p53 signaling pathway.

LncRNAs	Gene symbol	4	8	16	32	64 (J/m^2^)	Function
		Fold change					
GAS6-AS1	***GADD45A***	2.28	2.54	1.86	2.08	1.47	DNA damage, cell cycle, apoptosis
LOC338799							
GAS6-AS1	*ID3*	1.10	1.29	1.75	1.49	1.57	DNA damage
LOC644656							
GAS6-AS1	***TP53I3***	3.18	4.23	3.73	2.29	1.82	Cell death
LOC338799							
LOC644656							
GAS6-AS1	***MDM2***	3.49	3.35	3.30	2.26	1.21	Cell cycle arrest, apoptosis
LOC338799	***BAX***	1.71	2.4	2.45	2.46	1.99	Apoptosis
LOC338799	***CDKN1A***	2.28	3.56	3.04	2.50	1.95	Cell cycle arrest, apoptosis
LOC338799	*CDK6*	-1.03	-1.25	-1.96	-2.75	-3.46	G1 arrest
LOC338799	***ISCU***	2.63	3.17	3.62	4.76	4.10	-
LOC338799	***LCE1C***	3.92	7.72	6.48	9.92	5.15	DNA damage
LOC338799	***LCE1F***	6.01	9.96	14	20	13.6	DNA damage
USP17L6P	***TRIAP1***	3.74	6.56	8.69	18.5	13.4	Apoptosis
TERC	*MOAP1*	1.12	1.18	1.31	1.55	1.65	Cell death, apoptosis
TERC	*EIF3D*	1.12	1.47	1.51	1.94	1.99	Cell death, apoptosis

Genes in bold show more than 2-fold increase in expression levels in at least three groups.

## Data Availability

The data used to support the findings of this study are available from the corresponding author upon request.

## Supplementary Materials

Supplementary Materials Table S1: PCR primer sequences of genes and lncRNAs used in RT reaction and real-time reaction. Table S2: number of differentially expressed genes in UVC-radiated CD4 cells. Table S3: number of differentially expressed lncRNAs in UVC-radiated CD4 cells. Fig. S1: stem analysis of differentially expressed genes in UVC-radiated CD4 cells. Fig. S2: regression analysis of regulated genes and validation of lncRNA expression alterations. (a) Regression analyses of coexpressed genes TRIAP1, PLK2, NFKBIE, and ZPLD1 show dose-dependent relationships between expression alteration and the radiation dose within the range of 4-32 J/m2. (b) qRT-PCR results confirmed the expression alterations of CDKN1A1, GADD45A, TRIAP1, and PLK2 in CD4 cells at 24 h after UVC irradiation. ^∗^*P* < 0.05, ^∗∗^*P* < 0.01 compared with control group.
