# Supplementary material for: The Potential Regulatory Roles of lncRNAs in DNA Damage Response in Human Lymphocytes Exposed to UVC Irradiation
Source: Biomed Res Int. 2020 Mar 13;2020:8962635. doi: 10.1155/2020/8962635 (PMC7094206; doi:10.1155/2020/8962635)
Supplement: Supplementary Materials — Table S1: PCR primer sequences of genes and lncRNAs used in RT reaction and real-time reaction. Table S2: number of differentially expressed genes in UVC-radiated CD4 cells. Table S3: number of differentially expressed lncRNAs in UVC-radiated CD4 cells. Fig. S1: stem analysis of differentially expressed genes in UVC-radiated CD4 cells. Fig. S2: regression analysis of regulated genes and validation of lncRNA expression alterations. (a) Regression analyses of coexpressed genes TRIAP1, PLK2, NFKBIE, and ZPLD1 show dose-dependent relationships between expression alteration and the radiation dose within the range of 4-32 J/m2. (b) qRT-PCR results confirmed the expression alterations of CDKN1A1, GADD45A, TRIAP1, and PLK2 in CD4 cells at 24 h after UVC irradiation. ∗P < 0.05, ∗∗P < 0.01 compared with control group. [file 8962635.f1.docx]

Table S1 PCR primer sequences of genes and lncRNAs used in RT reaction and real-time reaction

| Gene Name |  | PCR primer sequence (5’-3’) |
| --- | --- | --- |
| *GADD45A* | Forward primer | TGCTGGTGACGAATCCACAT |
|  | Reverse primer | CCATCACCGTTCAGGGAGATT |
| *ISCU* | Forward primer | ACCAGTCAGCTGGGACATTT |
|  | Reverse primer | TACAAACGTGGGCTCCATGA |
| *LCE1F* | Forward primer | CTTGGGACTGACTGTGTTGC |
|  | Reverse primer | AGGCGCACAGATGGAATCA |
| *CDKN1A* | Forward primer | AGCAGAGGAAGACCATGTGG |
|  | Reverse primer | GGGTATGTACATGAGGAGGT |
| *PLK2* | Forward primer | CTACGCCGCAAAAATTATTCCTC |
|  | Reverse primer | TCTTTGTCCTCGAAGTAGTGGT |
| *TRIAP1* | Forward primer | CGACCTCTTCAAGCGCTACC |
|  | Reverse primer | CCCATGAACTCCAGTCCTTCAA |
| *GAPDH* | Forward primer | GGGAAACTGTGGCGTGAT |
|  | Reverse primer | GAGTGGGTGTCGCTGTTGA |
| GAS6-AS1 | Forward primer | GTGGGTACTGCATTCCTACCG |
|  | Reverse primer | CTCTCCTCTGATGGCAGGAC |
| TP53TG1 | Forward primer | ACGAAGGTACCCAACCCTCT |
|  | Reverse primer | GGTGTAAGTGTTCGCCTGGT |
| LOC338799 | Forward primer | GGGCAGGCCTAGTTTGACTC |
|  | Reverse primer | AGTCACTCCCCCTTCCAGTC |
| TERC | Forward primer | TGGCCATTTTTTGTCTAACCCTAAC |
|  | Reverse primer | TTTGCTCTAGAATGAACGGTGGAAG |

Table S2 Number of differentially expressed genes in UVC-radiated CD4 cells

| Categories | Number of up-regulated genes | | | | |  | Number of down-regulated genes | | | | |
| --- | --- | --- | --- | --- | --- | --- | --- | --- | --- | --- | --- |
|  | 4J/m² | 8J/m² | 16J/m² | 32J/m² | 64J/m² |  | 4J/m² | 8J/m² | 16J/m² | 32J/m² | 64J/m² |
| Fold≥2 | 125 | 248 | 381 | 489 | 502 |  | 24 | 105 | 481 | 759 | 1029 |
| Fold≥3 | 22 | 31 | 71 | 174 | 168 |  | 3 | 6 | 81 | 171 | 256 |
| Fold≥4 | 11 | 18 | 24 | 63 | 59 |  | 1 | 1 | 20 | 56 | 65 |
| Fold≥5 | 10 | 23 | 35 | 74 | 72 |  | 0 | 1 | 13 | 31 | 42 |
| Total | 168 | 320 | 511 | 800 | 801 |  | 28 | 113 | 595 | 1017 | 1392 |

Table S3 Number of differentially expressed lncRNAs in UVC-radiated CD4 cells

| Categories | Number of up-regulated lncRNAs | | | | |  | Number of down-regulated lncRNAs | | | | |
| --- | --- | --- | --- | --- | --- | --- | --- | --- | --- | --- | --- |
|  | 4J/m² | 8J/m² | 16J/m² | 32J/m² | 64J/m² |  | 4J/m² | 8J/m² | 16J/m² | 32J/m² | 64J/m² |
| Fold≥2 | 39 | 120 | 199 | 304 | 330 |  | 26 | 17 | 37 | 92 | 144 |
| Fold≥3 | 12 | 37 | 70 | 132 | 139 |  | 8 | 0 | 3 | 12 | 34 |
| Fold≥4 | 4 | 17 | 53 | 52 | 76 |  | 1 | 0 | 2 | 3 | 11 |
| Fold≥5 | 6 | 22 | 83 | 246 | 323 |  | 2 | 1 | 0 | 4 | 3 |
| Total | 61 | 196 | 405 | 734 | 868 |  | 37 | 18 | 42 | 111 | 192 |


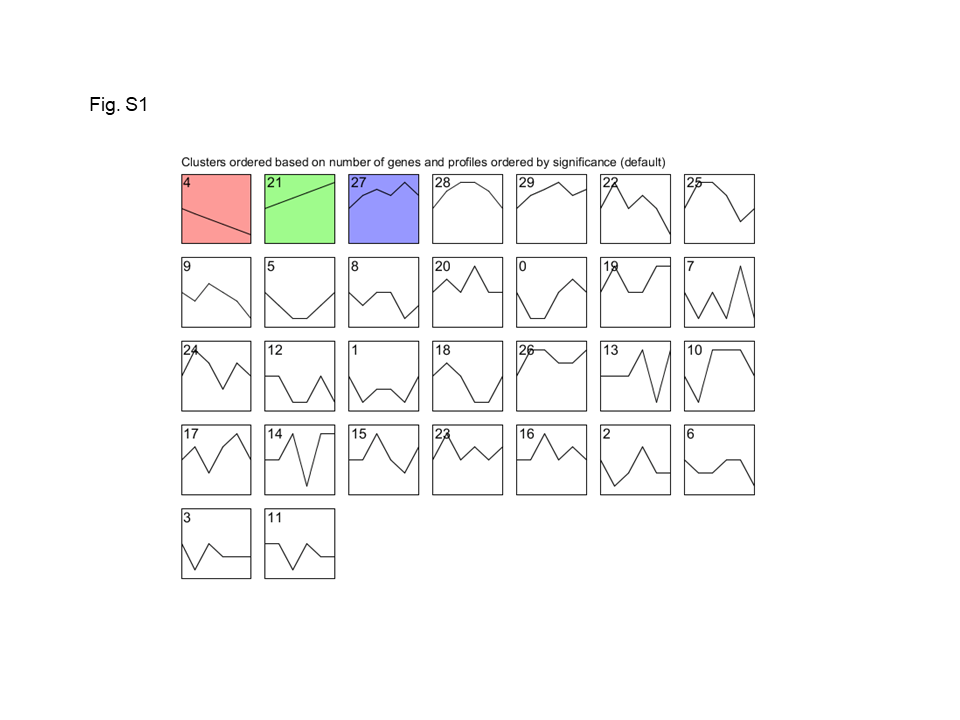


Fig. S1 Stem analysis of differentially expressed genes in UVC-radiated CD4 cells.


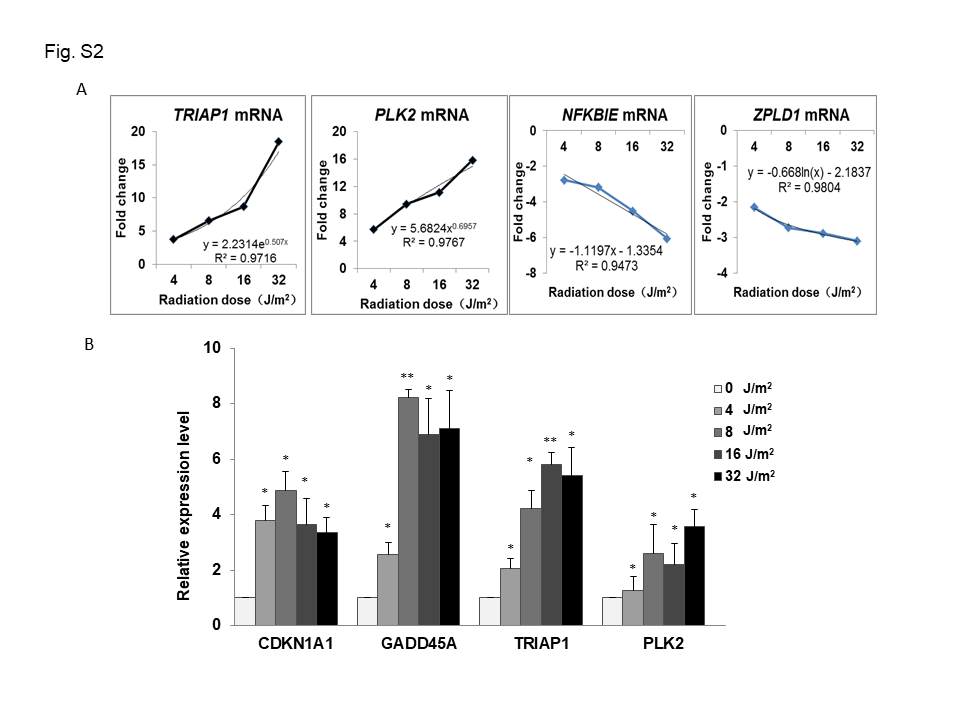


Fig. S2 Regression analysis of regulated genes and validation of lncRNA expression alterations. (A) Regression analyses of co-expressed genes TRIAP1, PLK2, NFKBIE and ZPLD1 show dose-dependent relationships between expression alteration and the radiation dose within the range of 4-32 J/m^2^. (C) qRT-PCR results confirmed the expression alterations of CDKN1A1, GADD45A, TRIAP1 and PLK2 in CD4 cells at 24 h after UVC irradiation. *P<0.05, **P<0.01 compared with control group.
